# Safety of core muscle training immediately after abdominal surgery: systematic review

**DOI:** 10.1093/bjsopen/zrad142

**Published:** 2023-12-18

**Authors:** Stéphanie F Perrodin, Lilian Salm, Guido Beldi

**Affiliations:** Department of Visceral Surgery and Medicine, Inselspital, Bern University Hospital, University of Bern, Bern, Switzerland; Graduate School for Health Sciences, University of Bern, Bern, Switzerland; Department of Visceral Surgery and Medicine, Inselspital, Bern University Hospital, University of Bern, Bern, Switzerland; Department of Pharmacology and Physiology, University of Calgary, Calgary, Alberta, Canada; Department of Visceral Surgery and Medicine, Inselspital, Bern University Hospital, University of Bern, Bern, Switzerland

## Introduction

Following abdominal surgery, abdominal wall-healing disorders are common, with up to 6% of patients developing fascial dehiscence (FD) and 35% incisional hernia (IH) within 2 years after high-risk surgery^1,2^.

A widely practised approach to avoid IH is restriction of postoperative activity, including avoidance of fascial strain and elevated intra-abdominal pressure through restriction of core muscle activity. Multiple studies highlight the considerable heterogeneity in instructions given to patients as to which activity can be resumed and when^3,4^. This resulted in recommendations based on the Delphi method or expert surveys, where avoidance of abdominal wall muscle strain was recommended for 2 weeks after laparoscopic and 4 weeks after open surgery^5,6^. Yet this is not supported by scientific evidence. In clinical research, there is evidence that coughing, vomiting and standing up, all unavoidable in the early postoperative phase, may generate higher intra-abdominal pressures compared to lifting or core muscle training^7,8^.

The aim of this review was to assess the safety of abdominal (core) muscle physiotherapy in terms of fascial healing, starting immediately after surgery involving an incision of the abdominal wall. The objective was to answer the following question: Does core muscle training immediately after abdominal surgery increase the risk of FD and/or IH?

## Methods

The authors conducted a systematic review according to the Cochrane guidelines and reported the findings according to the PRISMA guidelines^9,10^. The systematic review was not preregistered.

PubMed, CENTRAL and PEDro were searched on 30 September 2023. Studies investigating abdominal wall muscle exercises following abdominal surgery were included, starting on postoperative day one, and reporting on FD and/or IH. Two authors independently assessed inclusion criteria and biases and collected data. Details of the full methods, risk of bias assessment and statistical analysis can be found in the *Supplementary material*.

## Results

Two studies were included in the systematic review. *Figure S1* details the PRISMA flow diagram of study inclusion, and *Fig. S2* presents the risk of bias. Given the limited number of publications, a meta-analysis could not be performed.

### Study design and patient’s characteristics

The characteristics of the two RCTs can be found in *Table 1*^11,12^. The search yielded no studies that evaluated the safety of core muscle training in terms of FD and/or IH as primary end points.

**Table 1:** Characteristics of included studies

Study	Study design	Surgery	Exclusion criteria	Number of patients per arm	Comparator (standard of care)	Type of intervention	Primary outcome	Secondary outcomes	Follow-up
**Ahn *et al*., 2013^11^**	Single blind RCT, 1:1 randomization, minimization factors: age and gender	Open, laparoscopic or robotic surgery for stage I–III colon cancer	<20 or >70 years of age Recurrent or metastatic disease Immunosuppressants History of neoadjuvant chemotherapy or radiotherapy Use of ERAS protocol	*Control group:* *n* = 14 *Intervention group* *n* = 17	Unsupervised mobilization	Supervised exercise programme starting on POD 1 including core muscle training	Length of hospital stay	Time to flatus Anthropometric measures (body composition) Functional outcome measures (30 s chair stand test, timed single-leg stand test, Tecumseh step test) Complications: intraoperative complications, anastomotic leakage, iatrogenic bowel perforation, fascial dehiscence, ileus, wound infection, urine retention, renal failure, readmission	30 days
**De Almeida *et al*., 2017^12^**	Single blind RCT, 1:1 randomization	Elective major abdominal oncological surgery involving gastrointestinal or genitourinary tract with an expected duration >90 min	Pre-existing impairment of cardiac function, renal replacement therapy, acute kidney injury, venous thromboembolism, aortic dissection, thyrotoxicosis, inability to ambulate independently (6 min walk test), inability to exercise, bone metastasis, active infection, musculoskeletal and neurological conditions precluding exercise programme participation, palliative procedure	*Control group* *n* = 54 *Intervention group* *n* = 54	Supervised exercise programme starting on POD 1	Supervised exercise programme starting on POD 1 including core muscle training	Inability to cross the room or to walk 3 m without human assistance on POD 5 or at discharge	Functional walking capacity (6 min walk test) Incidence and intensity of fatigue (revised Piper fatigue scale) Health-related quality of life (EuroQol-5D-5L) Reduction in lean body mass (thigh circumference) Postoperative complications Exercise-related adverse events (falls, pain, dehiscence, syncope, postural hypotension)	30 days

POD, postoperative day.

The study population comprised 139 patients. Their characteristics including risk factors for FD or IH are presented in *Table S1* and details on patient inclusion are in the *Supplementary material*.

### Core muscle physiotherapy

*Table 2* presents the physiotherapy conducted in both studies. While the comparator in Ahn *et al*. was limited to unsupervised mobilization according to the patient’s capabilities^11^, the control group in De Almeida *et al*. participated in a programme where core control is listed as a physiotherapy element^12^. The exact components of core control are unclear and seem to be associated with the determination of core stability, defined as the ability to sit erect for at least 1 min. Nevertheless, it cannot be excluded that core training was also performed by the control group to some extent.

**Table 2:** Physiotherapy

Authors and year of publication	Study arm	Frequency	Duration of program	Supervision	Intensity	Core muscle exercises	Other exercises	Documentation of compliance and protocol deviation
**Ahn *et al*., 2013^11^**	Intervention	Two 15 min-sessions daily	From POD 1 to discharge	Phase 1: supervision by physiotherapist twice daily Phase 2 and 3: supervision by physiotherapist once daily	Step-up approach in three phases according to the patients capabilities	Phase 1: pelvic tilts (isometric contraction) Phase 2 and 3: pelvic tilts, pelvic thrust, one leg raise, crunch Isometric contractions held for 10 s	Stretching Resistance training (chest, shoulder, arm, thigh, calf). Up to 12 repetitions and 3 sets Balance training (one leg standing, one leg calf raise, hip adduction and abduction, hip flexion with knee bent, hip extension) Walking (unsupervised)	84.5% adherence to exercise programme Average number of sessions per phase: Phase 1: 1.18 Phase 2: 3.29 Phase 3: 5.75 5 patients discharged before reaching phase 3
	Control	Left up to the patient	From POD 1 to discharge	None	NA	Unsupervised walking in hallway	None	Mean total walking distance during hospitalization, in metres, documented
**De Almeida *et al*., 2017^12^**	Intervention	Two 30 min-sessions per day	From POD 1 to discharge	Supervision by physiotherapist twice daily	Step-up approach according to the patients' capabilities, after assessing core stability and muscle strength Use of perceived exertion scale for adapting exercise intensity	Core training: in a sitting position, trunk flexion and extension, lateral flexion and circles, repeated 10 times	Functional electrical stimulation, passive and active range of motion exercises for patients unable to perform resistance training Gait training: walking with or without walking aids. Increasing speed and decreasing reliance on aids Resistance training: isometric exercises of trunk, upper and lower extremities, each contraction lasting 3 s or more. 3 sets of 10 repetitions each. Isotonic training: with 1 or 2 kg weights, 3 sets of 10 repetitions each Aerobic training: cycle ergometer for 2 × 10 min or 20 min, not exceeding 80% of maximum heart rate	94.4% adherence to exercise programme Variation in intensity of exercise achieved by participants documented Protocol deviation: one patient started on POD 2, two patients did the exercises only once on one particular day
	Control	One 30-min session a day	From POD 1 to discharge	Supervision by physiotherapist	Unclear	Core control (not clearly defined)	Orthostatic training for patients with core control and sufficient lower extremity muscle strength Gait training (as above) Passive or active range of motion	Variation in intensity of exercise achieved by participants documented No protocol deviation

POD, postoperative day; NA, not available.

The exercises’ intensity and the number of repetitions varied according to the patient’s capabilities, variations that were not reported^11,12^.

### Fascial dehiscence and incisional hernia

No statistically significant difference in the occurrence of FD between the intervention and control groups was found, with only one event observed in the intervention group (*n* = 1/71, *P* = 0.335). The event was observed on the 28th postoperative day, following a surgical complication, and led to the patient’s death by refractory septic shock^12^. Ahn *et al*. reported no FD.

The authors found no study assessing the effect of core muscle exercises immediately after surgery on the development of IH. In the included studies, IH was not evaluated as an outcome, and the duration of follow-up would have been too short (30 days)^11,12^. A subgroup analysis was planned to account for the reported known risk factors for FD (see *Table S1*), but was not possible due to the low number of events.

### Other outcomes

In Ahn *et al.*, length of stay was significantly shorter in the intervention group than in the control group (*P* = 0.0005), yet no difference was found in Almeida *et al.* (*P* = 0.25)^11,12^. Further results including complications can be found in the *Supplementary material*.

## Discussion

Even with extended search criteria, the authors found only two publications, none of which was designed with IH or FD as primary end points. Thus, both are underpowered and lack sufficient follow-up time. The available evidence could speak towards the safety of abdominal wall muscle exercises immediately after surgery, as only one FD was reported, but the level of evidence is very low.

To assess the effect of physiotherapy on wound healing, findings need to be adjusted to the other known risk factors for FD and/or IH. In these publications, there was a lack of information on co-morbidities, with no significant differences reported, making it impossible to determine whether they played a role in the observed event^11,12^. Finally, it is unclear if the control group in De Almeida *et al*. also underwent core muscle exercises. If so, this would again speak towards the safety of such exercises, as no FD was observed in this group^12^.

This study highlights the need for further research in this domain. No evidence was found against core muscle exercises in the immediate postoperative phase, and a limited number of studies investigating this at all, even later in the recovery process^13,14^. In a similar systematic review on recovery after major abdominal wall repair, only one study was found that included physiotherapy but excluded lifting weights over 2.5 kg for 2 weeks after surgery^15,16^.

In animal studies, time to achieve 50% tensile strength of the rectus fascia after incision ranged from 3 to 8 days^17^. Generally, fascia healing seemed to be faster than dermis^18^, and the application of tension to the sutured fascia is thought to be beneficial, as mechanical strain seemed to stimulate fibroblast infiltration and collagen deposition and thus promote healing^19^. While this has yet to be proven in a clinical setting, it could be central to the promotion of abdominal wall exercises in the immediate postoperative phase.

Patients rely on their surgeons and general practitioners to help guide their recovery. Yet as demonstrated by this review, current recommendations are not evidence-based. We must be careful when limiting our patients' physical activity in the postoperative period, as it could have serious consequences in terms of quality of life and deconditioning.

This study is limited by the few publications found. Publication bias cannot be excluded, as studies that found core muscle exercises to have negative consequences for the patients in the immediate postoperative phase might not have been published. Finally, the authors are unable to make a statement as to whether these exercises influence the risk of IH, as this specific outcome has never been reported. The available evidence is insufficient to either recommend or discourage core muscle exercise immediately after abdominal surgery. There is currently only one ongoing RCT (NCT03808584^20^) investigating the effect of core muscle physiotherapy starting immediately after abdominal surgery on IH at 2 years. Based on these results and the results of this systematic review, further studies including co-morbidities for risk stratification and exploring different types and intensity levels of postoperative exercises are needed.

## Supplementary Material

zrad142_Supplementary_Data

## Data Availability

The data that support the findings of this study are available from the corresponding author upon reasonable request.
