# Supplementary material for: Safety of core muscle training immediately after abdominal surgery: systematic review
Source: BJS Open. 2023 Dec 18;7(6):zrad142. doi: 10.1093/bjsopen/zrad142 (PMC10726400; doi:10.1093/bjsopen/zrad142)
Supplement: zrad142_Supplementary_Data [file zrad142_supplementary_data.docx]

**Safety of core muscle training immediately after abdominal surgery: systematic review**

Stéphanie F. Perrodin^1,2^, Lilian Salm^1,3^, Guido Beldi^1^

^1^Department of Visceral Surgery and Medicine, Inselspital, Bern University Hospital, University of Bern, Bern, Switzerland

^2^Graduate School for Health Sciences, University of Bern, Switzerland

^3^ Department of Pharmacology and Physiology, University of Calgary, Calgary, Alberta, Canada

**Corresponding author:**

Prof. Dr. med. Guido Beldi, MD

Department of Visceral Surgery and Medicine

University Hospital Bern, Inselspital

Freiburgstrasse

3010 Bern

Switzerland

+41 31 632 82 75

guido.beldi@insel.ch

ORCID ID: [0000-0002-9914-3807](https://orcid.org/0000-0002-9914-3807)

Twitter: @BeldiGuido

**Supplementary Materials - Index**

| **Supplementary Methods** |  |
| --- | --- |
| 1. Eligibility criteria | *page 3* |
| 1. Outcome measures | *page 3* |
| 1. Search methods and study selection | *page 4* |
| 1. Data collection | *page 4* |
| 1. Assessment of risk of bias | *page 5* |
| 1. Statistical analysis | *page 5* |
| 1. Complete search strategy | *page 6* |
| **Supplementary Results** |  |
| 1. Study selection | *page 9* |
| 1. Study design | *page 9* |
| 1. Postoperative pain | *page 9* |
| 1. Length of stay | *page 9* |
| 1. Complications | *page 10* |
| 1. Risk of bias | *page 10* |
| **Supplementary Figures and Tables** |  |
| Figure S1: PRISMA flow diagram | *page 11* |
| Figure S2: Risk of Bias | *page 12* |
| Table S1: Patient’s characteristics | *page 13* |
| **References** | *page 14* |
|  |  |

**Supplementary Methods**

1. Eligibility criteria

The studies were included in the review if they met the following PICO (i.e. Patient, Intervention, Comparator, Outcome) criteria:

**Patient**: adults (18 years or older) undergoing abdominal surgery with an incision in the abdominal wall, be they laparoscopic or open procedures.

**Intervention**: postoperative physiotherapy including core muscle exercises focusing on the abdominal wall muscles, starting on postoperative day one at the latest.

**Comparator**: standard of care including no physiotherapy, respiratory physiotherapy or mobilization only, or delayed start of physiotherapy

**Outcome**: fascial dehiscence and/or incisional hernia.

Studies were excluded if: 1) the intervention consisted of pre-operative physiotherapy only, or where physiotherapy was limited to respiratory exercises or mobilization, 2) they included only patients after thoracic or cardiac surgery, 3) they focused on the ERAS (Enhanced Recovery After Surgery) protocols and did not include core muscle exercises, 4) core muscle exercises did not start immediately after surgery and 5) no results for the outcome of interest was reported (fascial dehiscence and/or incisional hernia).

We chose to include all prospective, retrospective and observational studies in this analysis and not limit the search to randomized controlled trials (RCTs), given the limited number of RCTs that we expected to find. Conference proceedings, unpublished reports such as preprints, and grey literature were excluded as the aim of this systematic review is to highlight the available scientific evidence as indexed in medical databases.

1. Outcome measures

The primary outcome was a composite endpoint of fascial dehiscence or incisional hernia in the first two years after surgery, according to the above-mentioned PICO framework.

The secondary outcomes included:

- Postoperative pain and its treatment
- Duration of stay
- Postoperative complications
- Adhesion to the exercise program and compliance, reasons for non-compliance
- Risk factors for fascial dehiscence or hernia

1. Search methods and study selection

A comprehensive literature search was initially conducted on 25 April 2021 and rerun on 15 February 2023, and again on 30 September 2023. The search strategy was developed by the first and second author. Previous, more limited searches yielded no results, which is why this search was made deliberately wide, to avoid excluding relevant studies.

MEDLINE (PubMed) (1967 to 30 September 2023), Embase (Elsevier) (1968 to 30 September 2023), Cochrane Central Register of Controlled Trials (CENTRAL) via the Cochrane Library (1970 to 30 September 2023) and PEDro were searched. Languages were limited to English, French and German. The reference lists of systematic reviews, randomized and non-randomized studies and editorials on similar topics were also examined (snowballing) ^1^. Furthermore, the ClinicalTrials.gov trial registration website (www.clinicaltrials.gov) was searched on 30 September 2023 for possibly relevant trials. If an eligible trial was marked as completed, the record of that study was tracked down. Finally, we also searched for retraction statements or errata for included studies.

The following search strategy combining MeSH terms and keywords were used: *abdominal surgery, laparotomy, laparoscopy, physiotherapy, exercise, rehabilitation, complication, hernia, fascial dehiscence*. The detailed search terms and search strategies for each database can be found in supplementary material 2.

All records were imported into Endnote^TM^ X20 (Clarivate, London, United Kingdom), where duplicates were removed.

SP and LS independently conducted the search, screened each record (title and abstract) for eligibility, and performed a detailed evaluation of full-text articles for inclusion. Discrepancies were settled by discussion and involvement of the senior author (GB).

1. Data collection

The following variables were extracted from the included studies by SP and LS:

- Study characteristics: author names, year of publication, study design, number of participants in each arms
- Participant characteristics: disease, age, risk factors for fascial dehiscence and incisional hernia including BMI and nutrition status, pulmonary complications, previous surgery, characteristics of surgery (open or laparoscopic, use of mesh, closure technique)
- Study intervention: type of core muscle exercises, compliance
- Primary endpoint: fascial dehiscence and incisional hernia
- Secondary endpoints: postoperative pain levels and treatment, other complications according to the Clavien-Dindo classification ^2^, surgical site infection, length of stay, duration and method of follow-up.

We chose not to report the outcomes describing physical performance, as they are beyond the scope of this review.

Attempts were made to contact the authors of the included studies (both corresponding authors and senior authors) for more information about the study populations and the variables but were without response.

1. Assessment of risk of bias

The Cochrane Collaboration’s tool for assessing risk of bias in randomized controlled trials (RoB2) was used independently by two authors (SP and LS) to assess the quality of the included RCTs ^3^. The following domains were evaluated: bias arising from the randomisation process, bias due to deviation from the intended intervention (effect of assignment and adherence to intervention), bias due to missing outcome data, bias in measurement of the outcome and bias in selection of the reported result. Because it is impossible to blind the participants and the physiotherapists to the intervention, this was not considered a risk of bias. The results are presented using the robvis visualisation Tool ^4^.

A funnel plot was planned to assess publication bias, but could not be done due to the low number of publications found ^5^. For the same reason, the overall certainty of evidence of the systematic review was not assessed ^6^.

1. Statistical analysis

A random-effects model was initially planned because of the different types of interventions in this review. Because of the limited number of studies investigating the evaluated intervention, the limited number of events and the low level of evidence, a meta-analysis could not be performed. The results of the studies included in this review were reported using descriptive statistics. These includes percentages, means and standard deviations (SD) and medians and inter-quartile ranges (IQR). Likewise, the possible sources of heterogeneity such as BMI, comorbidities, nutritional status, incision type and duration of follow-up would have been investigated with subgroup analysis should there have been sufficient publications included in the review. These results were also reported descriptively.

1. Complete search strategy

**Medline (PubMed)**

Search on the 15.02.2023, from 1967 to 30.09.2023, 14’002 records.

With limits adults, french, english, german: **7649 records**

| #23 | #5 AND #13 AND #17 AND #22 |
| --- | --- |
| #22 | #18 OR #19 OR #20 OR #21 |
| #21 | ("fascia"[MeSH Terms] OR "fascia"[All Fields] OR "fascial"[All Fields]) AND ("dehisce"[All Fields] OR "dehisced"[All Fields] OR "dehiscence"[All Fields] OR "dehiscences"[All Fields] OR "dehiscent"[All Fields] OR "dehisces"[All Fields] OR "dehiscing"[All Fields]) |
| #20 | "hernia"[MeSH Terms] OR "hernia"[All Fields] OR "hernias"[All Fields] OR "hernia s"[All Fields] OR "herniae"[All Fields] |
| #19 | ("adverse"[All Fields] OR "adversely"[All Fields] OR "adverses"[All Fields]) AND ("event"[All Fields] OR "event s"[All Fields] OR "events"[All Fields]) |
| #18 | "complicances"[All Fields] OR "complicate"[All Fields] OR "complicated"[All Fields] OR "complicates"[All Fields] OR "complicating"[All Fields] OR "complication"[All Fields] OR "complication s"[All Fields] OR "complications"[MeSH Subheading] OR "complications"[All Fields] |
| #17 | #14 OR #15 OR #16 |
| #16 | "inpatient s"[All Fields] OR "inpatients"[MeSH Terms] OR "inpatients"[All Fields] OR "inpatient"[All Fields] |
| #15 | "postoperative period"[MeSH Terms] OR ("postoperative"[All Fields] AND "period"[All Fields]) OR "postoperative period"[All Fields] OR "postop"[All Fields] OR "postoperative"[All Fields] OR "postoperatively"[All Fields] OR "postoperatives"[All Fields] |
| #14 | "perioperative"[All Fields] OR "perioperatively"[All Fields] |
| #13 | #6 OR #7 OR #8 OR #9 OR #10 OR #11 OR #12 |
| #12 | "recoveries"[All Fields] OR "recovery"[All Fields] |
| #11 | "rehabilitant"[All Fields] OR "rehabilitants"[All Fields] OR "rehabilitate"[All Fields] OR "rehabilitated"[All Fields] OR "rehabilitates"[All Fields] OR "rehabilitating"[All Fields] OR "rehabilitation"[MeSH Terms] OR "rehabilitation"[All Fields] OR "rehabilitations"[All Fields] OR "rehabilitative"[All Fields] OR "rehabilitation"[MeSH Subheading] OR "rehabilitation s"[All Fields] OR "rehabilitational"[All Fields] OR "rehabilitator"[All Fields] OR "rehabilitators"[All Fields] |
| #10 | "education"[MeSH Subheading] OR "education"[All Fields] OR "training"[All Fields] OR "education"[MeSH Terms] OR "train"[All Fields] OR "train s"[All Fields] OR "trained"[All Fields] OR "training s"[All Fields] OR "trainings"[All Fields] OR "trains"[All Fields] |
| #9 | ("exercise"[MeSH Terms] OR "exercise"[All Fields] OR "exercises"[All Fields] OR "exercise therapy"[MeSH Terms] OR ("exercise"[All Fields] AND "therapy"[All Fields]) OR "exercise therapy"[All Fields] OR "exercise s"[All Fields] OR "exercised"[All Fields] OR "exerciser"[All Fields] OR "exercisers"[All Fields] OR "exercising"[All Fields]) AND ("program"[All Fields] OR "program s"[All Fields] OR "programe"[All Fields] OR "programed"[All Fields] OR "programes"[All Fields] OR "programing"[All Fields] OR "programmability"[All Fields] OR "programmable"[All Fields] OR "programmably"[All Fields] OR "programme"[All Fields] OR "programme s"[All Fields] OR "programmed"[All Fields] OR "programmer"[All Fields] OR "programmer s"[All Fields] OR "programmers"[All Fields] OR "programmes"[All Fields] OR "programming"[All Fields] OR "programmings"[All Fields] OR "programs"[All Fields]) |
| #8 | "exercise"[MeSH Terms] OR "exercise"[All Fields] OR "exercises"[All Fields] OR "exercise therapy"[MeSH Terms] OR ("exercise"[All Fields] AND "therapy"[All Fields]) OR "exercise therapy"[All Fields] OR "exercise s"[All Fields] OR "exercised"[All Fields] OR "exerciser"[All Fields] OR "exercisers"[All Fields] OR "exercising"[All Fields] |
| #7 | "exercise"[MeSH Terms] OR "exercise"[All Fields] OR ("physical"[All Fields] AND "activity"[All Fields]) OR "physical activity"[All Fields] |
| #6 | "physical therapy modalities"[MeSH Terms] OR ("physical"[All Fields] AND "therapy"[All Fields] AND "modalities"[All Fields]) OR "physical therapy modalities"[All Fields] OR "physiotherapies"[All Fields] OR "physiotherapy"[All Fields] |
| #5 | #1 OR #2 OR #3 OR #4 |
| #4 | “laparoscopie"[All Fields] OR "laparoscopy"[MeSH Terms] OR "laparoscopy"[All Fields] OR "laparoscopies"[All Fields] |
| #3 | "laparotomy"[MeSH Terms] OR "laparotomy"[All Fields] OR "laparotomies"[All Fields] |
| #2 | ("visceral"[All Fields] OR "viscerally"[All Fields]) AND ("surgery"[MeSH Subheading] OR "surgery"[All Fields] OR "surgical procedures, operative"[MeSH Terms] OR ("surgical"[All Fields] AND "procedures"[All Fields] AND "operative"[All Fields]) OR "operative surgical procedures"[All Fields] OR "general surgery"[MeSH Terms] OR ("general"[All Fields] AND "surgery"[All Fields]) OR "general surgery"[All Fields] OR "surgery s"[All Fields] OR "surgerys"[All Fields] OR "surgeries"[All Fields]) |
| #1 | ("abdominal"[All Fields] AND "surgery"[All Fields]) OR "abdominal surgery"[All Fields] |

**Embase**

search on the 30.09.2023, from 1968 to September 2023, 4693 records

With limits: adults: **2494** records.

PICO search
('abdominal surgery'/exp OR 'abdomen surgery' OR 'abdominal operation' OR 'abdominal surgery' OR 'digestive system surgery' OR 'digestive system surgical procedures' OR 'surgery, abdominal' OR 'upper abdomen surgery' OR 'laparotomy'/exp OR 'abdomen laparotomy' OR 'abdominal laparotomy' OR 'laparotomy' OR 'laparoscopy'/exp OR 'laparoscopy' OR 'laparoscopy, video' OR 'pelvic endoscopy' OR 'peritoneoscopy' OR 'video laparoscopy' OR 'videolaparoscopy' OR 'laparoscopic' OR 'gastrectomy'/exp OR 'gastrectomy' OR 'gastric resection' OR 'gastroresection' OR 'hemigastrectomy' OR 'resection, gastric' OR 'stomach extirpation' OR 'stomach resection' OR 'stomach transection' OR 'stomach surgery'/exp OR 'gastric surgery' OR 'stomach operation' OR 'stomach surgery' OR 'stomach suturing' OR 'esophagus resection'/exp OR 'esophageal resection' OR 'esophagectomy' OR 'esophagus resection' OR 'esophagus shortening' OR 'oesophageal resection' OR 'oesophagectomy' OR 'oesophagus resection' OR 'resection, esophagus' OR 'liver surgery'/exp OR 'hepatic surgery' OR 'liver operation' OR 'liver surgery' OR 'surgery, liver' OR 'liver resection'/exp OR 'hepatectomy' OR 'hepatic resection' OR 'liver resection' OR 'resection, liver' OR 'cholecystectomy'/exp OR 'cholecystectomy' OR 'gallbladder resection' OR 'pancreatectomy'/exp OR 'hemipancreatectomy' OR 'pancreas resection' OR 'pancreatectomy' OR 'pancreatectomy total' OR 'total pancreatectomy' OR 'colorectal surgery'/exp OR 'colon and rectal surgery (speciality)' OR 'colon and rectal surgery (specialty)' OR 'coloproctotomy' OR 'colorectal surgery' OR 'proctocolonic surgery' OR 'surgery, colorectal' OR 'bariatric surgery'/exp OR 'bariatric operation' OR 'bariatric operations' OR 'bariatric procedure' OR 'bariatric procedures' OR 'bariatric surgery' OR 'bariatric surgical procedure' OR 'bariatric surgical procedures' OR 'metabolic surgery' OR 'obesity operation' OR 'obesity surgery' OR 'obesity surgical treatment' OR 'surgery, bariatric' OR 'weight loss operation' OR 'weight loss surgery' OR 'weight reduction operation' OR 'weight reduction surgery') AND ('physiotherapy'/exp OR 'physical therapy' OR 'physical therapy (speciality)' OR 'physical therapy (specialty)' OR 'physical therapy modalities' OR 'physical therapy service' OR 'physical therapy speciality' OR 'physical therapy specialty' OR 'physical therapy techniques' OR 'physical treatment' OR 'physio therapy' OR 'physiotherapy' OR 'physiotherapy department' OR 'therapy, physical' OR 'exercise'/exp OR 'biometric exercise' OR 'exercise' OR 'exercise training' OR 'fitness training' OR 'fitness workout' OR 'physical conditioning, human' OR 'physical effort' OR 'physical exercise' OR 'physical work-out' OR 'physical workout' OR 'rehabilitation'/exp OR 'rehabilitation' OR 'rehabilitation program' OR 'rehabilitation programme') AND ('postoperative period'/exp OR 'postoperative period' OR 'postoperative phase' OR 'perioperative period'/exp OR 'perioperative care' OR 'perioperative period' OR 'postsurgical' OR 'inpatient') AND ('complication'/exp OR 'complication' OR 'complications' OR 'hernia'/exp OR 'hernia' OR 'herniation' OR 'fascial dehiscence'/exp OR 'adverse event')

**PEDro**

search on the 30.09.2023

Search 1: abdominal surger*: **266 records**

Search 2: postoperative physiotherapy **360 records**

**Central**

Search on the 30.09.2023, from 1970 to 2023, **4033 records**

#1 abdominal surgery 21499

#2 visceral surgery 1856

#3 laparotomy 4283

#4 laparoscop* 27150

#5 #1 OR #2 OR #3 OR #4 46754

#6 physiotherapy 21814

#7 exercise 129980

#8 exercise programme 37706

#9 rehabilitation 80325

#10 recovery 77952

#11 #6 OR #7 OR #8 OR #9 OR #10 252512

#12 perioperative 26679

#13 postoperative 151246

#14 inpatient 16684

#15 #12 OR #13 OR #14 8105

#16 complication* 231242

#17 adverse event* 169985

#18 hernia 8105

#19 fascial dehiscence 133

#20 #16 OR #17 OR #18 OR #19 377327

#21 #5 AND #11 AND #15 AND #20 4033

**ClinicalTrials.gov**

Search on the 30.09.2023

Condition: surgery

Other terms: postoperative physiotherapy OR postoperative exercise

**Supplementary Results**

1. Study selection

Figure S1 details the PRISMA flow diagram of study inclusion. On ClinicalTrials.gov, only one study (NCT03808584) met the inclusion criteria. The results are not available as the study is still recruiting.

1. Study design

Both studies calculated sample sizes according to their primary endpoints, which was length of stay^7^ and inability to walk 3 meters at the 5^th^ postoperative day^8^. Ahn et al initially planned 26 patients per arm, but due to the introduction of the ERAS protocol in the study centre enrolment was stopped at 20 patients per arms ^7^. Three patients were excluded after randomisation due to a change in primary diagnosis in the control group and two in the intervention group. Three patients dropped-out in the control group and two in the intervention group, leaving 14 patients in the control group and 17 patients in the intervention group for analysis ^7^. De Almeida et al included 108 patients in their study, 54 per arm, and reported no dropouts or deviation of allocation ^8^. In both of the included studies, the physiotherapists and patients could not be blinded to the intervention because of its nature, but the outcome assessors were blinded^7,8^.

1. Postoperative pain

In De Almeida et al, pain was observed at the time of exercise in 20.4 % (11/54) of patients in the control group and 27.8 % (15/54) of patients in the intervention group. Both studies described adapting the physiotherapy program to the patients’ pain level ^7,8^. There is no further information as to which exercises caused pain, what the pain intensity was and what measures were taken ^8^.

1. Length of stay

Ahn et al reports length of hospital stay as its primary outcome, which was significantly lower in the intervention group, with a mean of 7.82 days (SD 1.07 days) in the intervention group versus 9.86 days (SD 2.66 days) in the control group (p = 0.005).

In De Almeida et al, length of stay was documented as a secondary outcome, which was comparable in both groups, with a median of 8 days (IQR 6-13) in the intervention group versus 8 days (IQR 7-13) in the control group (p = 0.25).

1. Complications

Ahn et al reports two minor complications, one case of surgical site infection in the intervention group and one case of postoperative ileus in the control group. There were no readmissions or reoperation during the 30-day follow-up period ^7^.

De Almeida et al reports 48 minor complications (Clavien-Dindo ≤ IIIa) in the intervention group and 46 in the control group. Major complications (Clavien-Dindo > IIIa) accounted for 11.1% (6/54) of complications in the intervention group and 14.8% (8/54) in the control group. Two patients in the intervention group died due to refractory septic shock, one after obstructive acute abdomen, the other of the above-mentioned fascial dehiscence. Two patients died after discharge in the control group, the cause of death is unknown. Surgical site infections occurred in 3.7 % (2/54) of patients in the intervention group and 9.3 % (5/54) in the control group. Among the physiotherapy-specific complications, they observed no falls nor syncope, and postural hypotension in 18.5 % (10/54) of patients in the control group and 24.1 % (13/54) in the control group. The other complications are not described ^8^.

1. Risk of bias

The risk of bias according to the RoB2 assessment tool is illustrated in Figure S2. Globally, the risk of bias for both studies is assessed as high. Publication bias could not be assessed.

**Supplementary Figures and Tables**

**
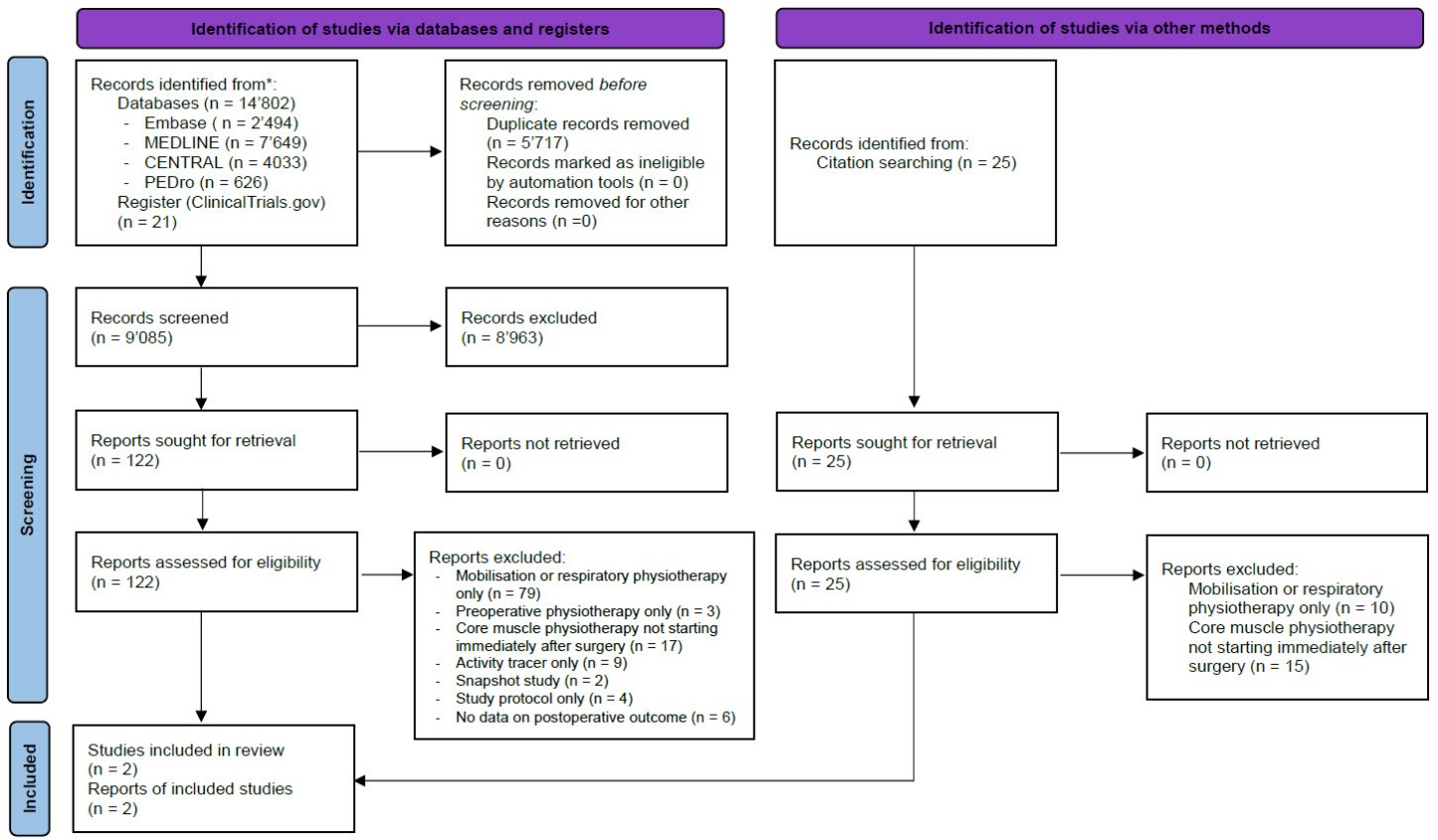
**

**Figure S1:** PRISMA flow diagram of included studies. Adapted from Page et al ^9^.


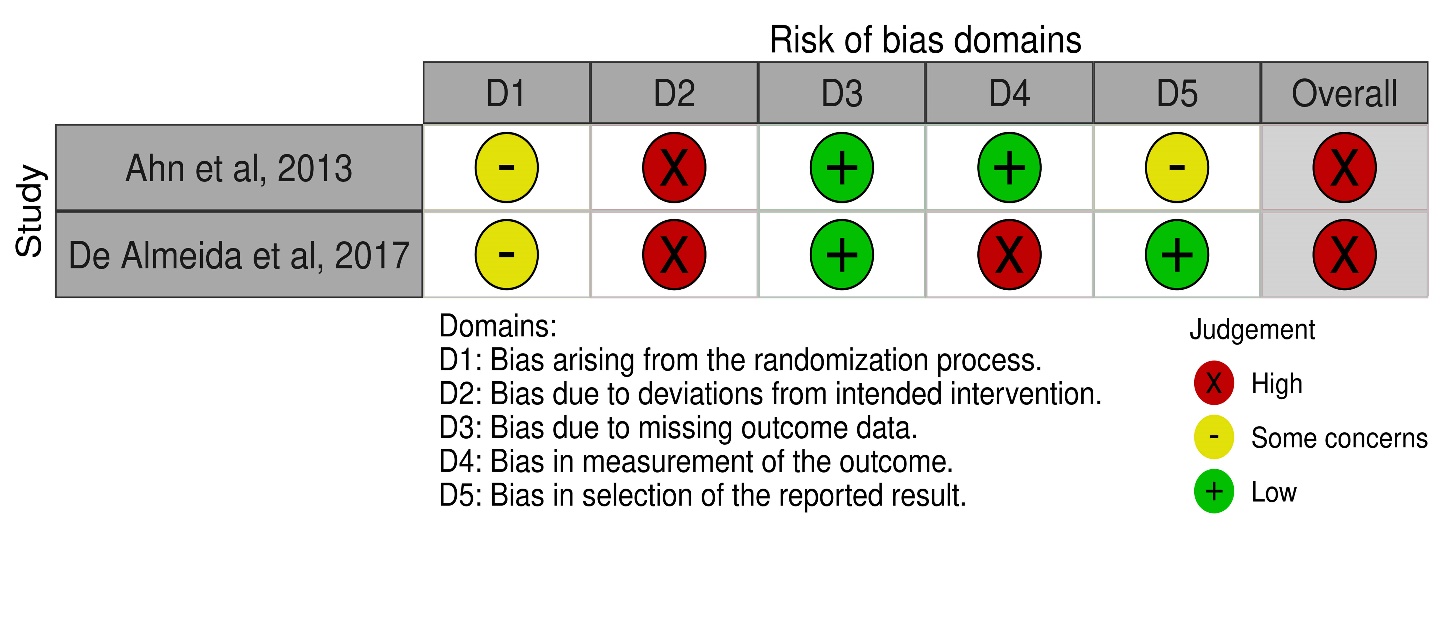


**Figure S2**: results of the risk of bias analysis using the robvis RoB2 visualisation Tool ^4^.

| **Table S1: Patients’ characteristics** | | | | | | | | | | | |
| --- | --- | --- | --- | --- | --- | --- | --- | --- | --- | --- | --- |
| **study** | **study arm** | **number of patients** | **age (years)** | **sex (male)** | **BMI (kg/m^2^)** | **ASA score < III** | **pulmonary comorbidities** | **previous surgery** | **nutritional risk screening score <3** | **open surgery** | **closure technique** |
| Ahn, 2013 ^7^ | intervention  control | 17  14 | 55.61 ± 7.11*  57.43 ± 6.12*  p = 0.453^†^ | 70.6% (12/17)  35.7% (5/14)  p = 0.076^‡^ | 22.6 ± 3.01*  24.3 ± 3.4*  p = 0.153^†^ | 100% (17/17)  100% (14/14)  p = 1.00^‡^ | NA | NA | NA | 11.8% (2/17)  21.4% (3/14)  p = 0.434 | NA |
| De Almeida, 2017 ^8^ | intervention  control | 54  54 | 61 (53-70)°  62 (51-68)°  p = NA | 38.9% (21/54)  40.7% (22/54)  *p = 0.844^¥^* | 25 (IQR 23-28)°  25 (IQR 22-27)°  p = NA | 70.4% (38/54)  85.2% (46/54)  *p = 0.064^¥^* | 3.7% (2/54)  3.7% (2/54)  *P=1.000^¥^* | NA | 62.9% (34/54)  68.5% (37/54)  *p = 0.543^¥^* | 79.6% (43/54)  75.9% (41/54)  *p = 0.643^¥^* | NA |

Results: * mean ± SD, ° median (IQR)

statistical analysis: ^†^ t-test, ^‡^ Fischer’s exact test, ^¥^ Chi-square test. The statistical test conducted by the review authors (not available from the publication) are in italic.

*ASA* American Society of Anaesthesiology score*, IQR* Inter-Quartile Range, *NA* not available, *SD* Standard Deviation

**References**

1. Greenhalgh T, Peacock R. Effectiveness and efficiency of search methods in systematic reviews of complex evidence: audit of primary sources. Bmj. 2005;331(7524):1064-1065.

2. Dindo D, Demartines N, Clavien PA. Classification of surgical complications: a new proposal with evaluation in a cohort of 6336 patients and results of a survey. Ann Surg. 2004;240(2):205-213.

3. Sterne JAC, Savović J, Page MJ, Elbers RG, Blencowe NS, Boutron I, et al. RoB 2: a revised tool for assessing risk of bias in randomised trials. Bmj. 2019;366:l4898.

4. McGuinness LA, Higgins JPT. Risk-of-bias VISualization (robvis): An R package and Shiny web app for visualizing risk-of-bias assessments. Research Synthesis Methods. 2020;n/a(n/a).

5. Egger M, Davey Smith G, Schneider M, Minder C. Bias in meta-analysis detected by a simple, graphical test. Bmj. 1997;315(7109):629-634.

6. Guyatt G, Oxman AD, Akl EA, Kunz R, Vist G, Brozek J, et al. GRADE guidelines: 1. Introduction-GRADE evidence profiles and summary of findings tables. J Clin Epidemiol. 2011;64(4):383-394.

7. Ahn KY, Hur H, Kim DH, Min J, Jeong DH, Chu SH, et al. The effects of inpatient exercise therapy on the length of hospital stay in stages I-III colon cancer patients: Randomized controlled trial. International Journal of Colorectal Disease. 2013;28(5):643-651.

8. de Almeida EPM, de Almeida JP, Landoni G, Galas F, Fukushima JT, Fominskiy E, et al. Early mobilization programme improves functional capacity after major abdominal cancer surgery: a randomized controlled trial. British journal of anaesthesia. 2017;119(5):900-907.

9. Page MJ, McKenzie JE, Bossuyt PM, Boutron I, Hoffmann TC, Mulrow CD, et al. The PRISMA 2020 statement: An updated guideline for reporting systematic reviews. J Clin Epidemiol. 2021;134:178-189.
